# The use of multi-gap resistive plate chambers for in-beam PET in proton and carbon ion therapy

**DOI:** 10.1093/jrr/rrt042

**Published:** 2013-07

**Authors:** David Watts, Giacomo Borghi, Fabio Sauli, Ugo Amaldi

**Affiliations:** 1 TERA Foundation, 11, Via Puccini, I-28100, Novara, Italy; 2 Universita Autonoma de Barcelona, Bellaterra, Spain

**Keywords:** in-beam PET, resistive plate chambers, on-line dosimetry, hadron therapy, quality assurance

## Abstract

On-line verification of the delivered dose during proton and carbon ion radiotherapy is currently a very desirable goal for quality assurance of hadron therapy treatment plans. In-beam positron emission tomography (ibPET), which can provide an image of the β+ activity induced in the patient during irradiation, which in turn is correlated to the range of the ion beam, is one of the modalities for achieving this goal. Application to hadron therapy requires that the scanner geometry be modified from that which is used in nuclear medicine. In particular, PET detectors that allow a sub-nanosecond time-of-flight (TOF) registration of the collinear photons have been proposed. Inclusion of the TOF information in PET data leads to more effective PET sensitivity. Considering the challenges inherent in the ibPET technique, namely limited β+ activity and the effect of biological washout due to blood flow, TOF-PET technologies are very attractive. In this context, the TERA Foundation is investigating the use of resistive plate chambers (RPC) for an ibPET application because of their excellent timing properties and low cost. In this paper we present a novel compact multi-gap RPC (MRPC) module design and construction method, which considering the large number of modules that would be needed to practically implement a high-sensitivity RPC-PET scanner, could be advantageous. Moreover, we give an overview of the efficiency and timing measurements that have been obtained in the laboratory using such single-gap and multi-gap RPC modules.

## INTRODUCTION

On-line *in vivo* verification of the delivered dose during proton and carbon ion beam therapy is currently a very desirable goal for quality assurance in clinical hadron therapy. The use of radiation detectors has been proposed for this purpose. Several detection modalities can be exploited in which information about the volumetric delivered dose is obtained by detecting secondary particles escaping the body. These secondary particles are produced either promptly during irradiation, or by the decay of radioactive isotopes produced within the patient tissue by the therapeutic beam.

Currently, the detection of the two co-linear 511 keV gamma rays caused by the decay of β+ emitters induced along the beam path is the most promising modality for *in vivo* dosimetry of proton and ion-beams [1]. This modality requires the use of detector technologies similar to the ones used in positron emission tomography (PET). Reconstruction of the volumetric concentration of β+ emitters produced within the body by the therapeutic irradiation has been shown to yield accurate information about the range of the ion beam in the patient.

The technique, known as in-beam PET (ibPET), has been used clinically at both the Heavy Ion Medical Accelerator in Chiba (Japan) [2] and the Gessellschaft fur Schwerionenforschung Darmstadt (GSI) in Europe. In the GSI study between 1997 and 2008, ∼ 430 patients were monitored with a custom-built dual-head PET scanner, named BASTEI, directly following treatment with carbon ions. Deviations between the β+ concentration predicted by simulation and the measured one were used to check the range distribution of the stopped carbon ions. The ibPET data was used to track errors between the expected and delivered treatment plan caused by patient misalignment, organ motion and density changes due to cavity filling within the patient [3].

Although the GSI study concluded that in-beam PET can provide useful clinical information about the delivered dose *in vivo*, it also highlighted the challenges, namely the low statistics (caused by the limited number of β+ isotopes induced in the patient and the limited angular field-of-view (FOV) of the dual-head geometry) and the effect of washing-out of the β+ concentration due to blood flow in the minutes following treatment. Since the time of the pilot study at GSI, there has been a continuing interest in developing a new in-beam PET scanner that would improve upon the performance of the BASTEI scanner.

In light of the special challenges inherent in ibPET the most important parameter that must be considered is the final scanner sensitivity [4]. Since increasing the angular and axial FOV beyond the dimensions of the BASTEI necessarily implies a substantial increase in material cost, current research favors the use of new technologies that increase the scanner's ‘effective’ sensitivity in other ways.

A hot topic in mainstream PET research for nuclear medicine is the use of PET technologies capable of measuring the time-of-flight (TOF) of the coincident and co-linear 511-keV gamma rays. Conventional PET scanners register only the detection of coincident photons within a rather large time window (several nanoseconds). A TOF measurement with sub-nanosecond precision effectively translates into a higher PET sensitivity, since the origin of the positron annihilation can be confined to a region along the line-of-response (LOR) rather than anywhere along the LOR [5]. Considering the challenges for ibPET, a TOF-capable scanner having a very high coincidence TOF resolution is very attractive.

Already, commercial TOF-PET scanners are being used for off-line *in vivo* dosimetry in hadron therapy, acquiring data in the minutes immediately after therapy [6]. This solution avoids having to build a custom scanner of limited angular FOV, and allows to take advantage of the commercial PET market. Its drawbacks, however, are loss of activity contribution of the short-lived β+ emitters and the need for patient re-positioning after treatment.

The AQUA (Advanced QUality Assurance) group of the TERA Foundation has been studying novel PET detector designs that are TOF-capable and scalable to large-coverage PET geometries. In particular, resistive plate chambers (RPC) have been studied. Though typically not used for gamma detection, RPCs are enticing for a PET application because of their excellent timing resolution for tracking charged particles, and because they are very cheap to produce in large surface areas [7].

Popular in high-energy physics instrumentation, the main drawback of RPCs for PET is their very low detection efficiency for 511 keV photons. In most RPC applications, the goal is to detect charged particles, which create a trail of ionizations within the gas volume upon their passage. In an RPC-PET detector, however, the detection principle is different: the gamma rays interact in the solid electrodes, and if an energetic electron produced by this interaction reaches the gas gap it can be multiplied and produce a signal.

Detection of a gamma ray in an RPC requires that an energetic electron produced within the bulk of an electrode escapes into the gas volume with enough energy to initiate avalanche multiplication. The detection efficiency depends on the conversion efficiency of the electrode material and the limited range of the electron in that material. Since most RPC designs make use of soda-lime float glass it follows that a single RPC is inefficient for gamma ray conversion at the characteristic energy of 511 keV. Denser materials such as lead-glass have been suggested, but so far obtaining samples of thin sheets of such materials at a reasonable cost has excluded this possibility. The multi-gap RPC (MRPC), consisting of many electrodes separating several gas-gaps read out as a single detector, can provide a solution.

MRPCs are already widely used in high-energy physics experiments with excellent timing resolutions: a 20 ps full width at half maximum (FWHM) has been achieved for a 24-gap chamber tracking charged particles [8]. To date, however, their application to PET has been uncertain, the main challenge being their low efficiency. A further complication is that for gamma rays, only a single gap of the MRPC will fire at one time, resulting in a weak signal being induced on the electrodes. It is expected that this will degrade the timing somewhat from the values obtained in particle-tracking applications. That being said, several research groups are actively studying the use of RPCs for nuclear medicine [9–11], particularly for full-body 3D PET imaging [12]. The hope is that despite the challenges, their very low cost will make it feasible to stack large numbers of MRPC modules with large surface area, thus increasing the efficiency. If the total efficiency can be made similar to that of crystal-based technologies, then the excellent timing and spatial resolution properties of MRPCs could be exploited to make an economical PET scanner with very good performance characteristics.

In order to investigate their performance for an ibPET application, and equivalently for a regular PET application for nuclear medicine, the AQUA group has constructed a number of MRPC prototypes and tested their performance in the laboratory using a ^22^Na source, as a β+ emitter. These prototype designs will be described along with the measured detection efficiency of single-gap and MRPC modules. We also present a preliminary timing measurement obtained with two identical detectors placed in a coincidence setup.

## MATERIALS AND METHODS

**Fig. 1.**
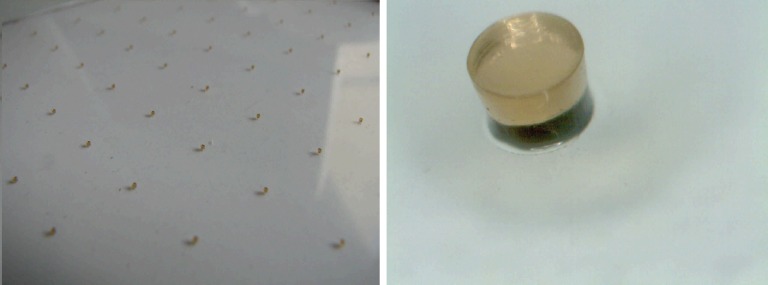
RPC spacers produced from photo-sensitive polyimide. Each spacer is 300 µm in diameter and 300 µm in height.

In most MRPC designs for high-energy physics, nylon fishing lines are used as a mechanical spacer between electrodes [13, 14]. Instead, we use a photo-sensitive polyimide film common in the printed circuit board (PCB) industry. By photo-lithographic means, the polyimide film is first laminated onto the glass and then selectively etched in any image desirable. We have used a pattern of spacers of 300-µm diameter and 300-µm height covering the glass surface at 1-cm separation (see Fig. 1). In this way, excess mechanical support—needed to fix the fishing line—can be avoided, allowing for a very compact design that is also easier to assemble.

**Fig. 2.**
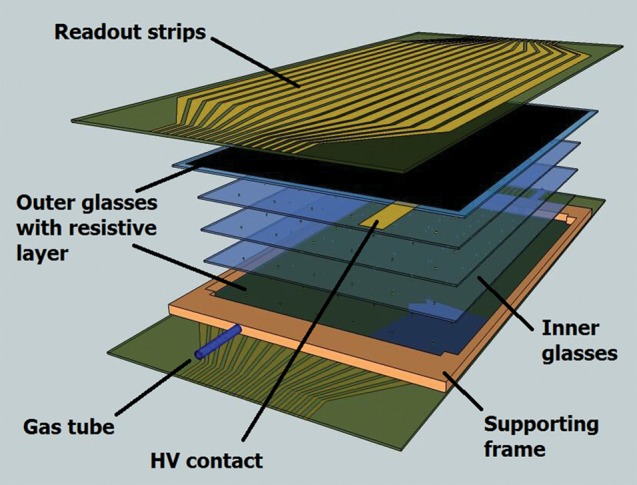
Schematic of compact MRPC design for TOF-PET application.

A schematic of an MRPC design for a PET application is shown in Fig. 2. The assembly consists of glass plates glued inside a very low-density glass-epoxy composite frame. The outmost glasses are coated with a resistive layer that allows application of a high voltage (HV). A pair of strip-readout electrodes patterned onto flexible polyimide foils are placed above the resistive layers, bringing the induced signal to the front-end electronics. The pitch between readout strips has been chosen to be 4 mm, roughly similar to the segmentation of most crystal-based PET designs.

One aspect of importance to the operation of RPCs is the value of the surface resistivity of the coating needed to apply the HV over the active area, typically measured in Ω/square. In our case, a higher resistivity is desirable since the less resistive the layer is, the more the signal produced inside the detector is spread out over the readout electrodes, resulting in a lower signal on each channel. Since we can assume that less overall charge will be induced on the electrodes when detecting 511-keV photons (only one gap fires rather than many as with charged particles), it follows that too low a resistivity will limit the detection efficiency. This point has been investigated with several types of materials. A resistivity of 1 MΩ/square, which was about the highest that could be achieved while still remaining uniform over the entire active area, was deemed suitable for our geometry. The layer is a colloidal graphite emulsion that is first applied to the glass surface and then allowed to dry, forming a thin resistive layer. Other materials that offer a resistivity higher than 1 MΩ/square are currently under investigation.

**Fig. 3.**
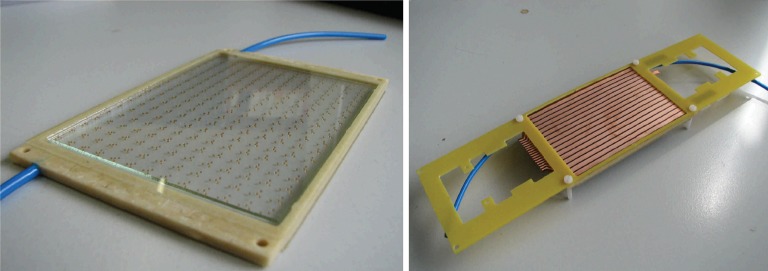
A 4-gas-gap MRPC module having 6.5 cm × 9 cm active area, shown before the resistive coating is applied (left), and after the full assembly (right).

We have constructed prototype MRPC modules, having an active area of 6.5 cm × 9 cm, and tested them for gas tightness and HV stability. One example of a 4-gas-gap module is shown in Fig. 3, both before resistive coating was applied (shown left) and after the assembly was complete with the resistive coating, readout strips and front-end electronics support (shown right). Such modules use 400-µm soda-lime glass and 300-µm spacers and are only 3.2 mm thick, making it possible to stack many tens of modules into a single and compact PET camera head.

**Fig. 4.**
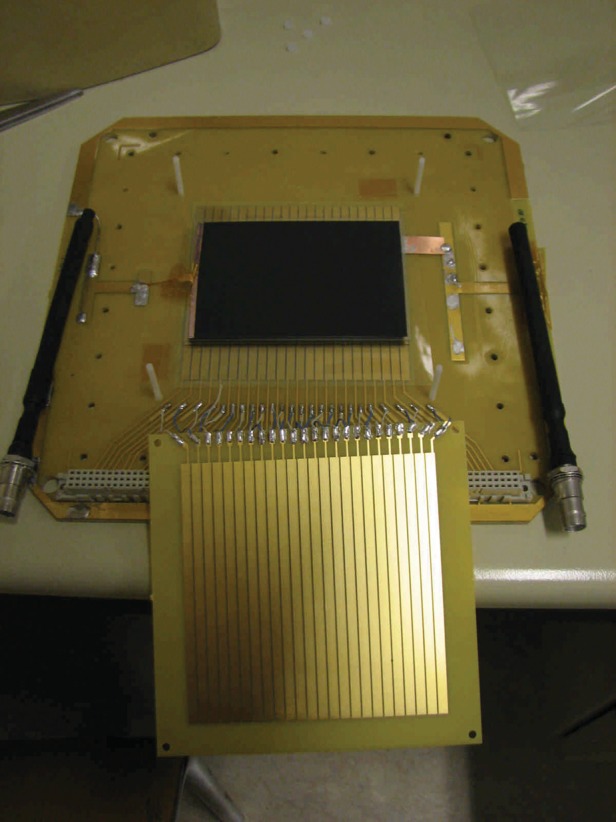
An MRPC mounted inside the experimental gas chamber. The upper readout strips have not yet been mounted in order to show the resistive colloidal graphite layer used to distribute the HV over the detector area.

Single-gap and MRPC prototypes, similar to the ones shown in Fig. 3, were tested for their efficiency and timing properties in the laboratory. Instead of choosing to use glued modules, however, we have built RPCs using the same materials and techniques as before but housed inside larger gas chambers. These gas chambers can be disassembled easily, allowing us more flexibility in choosing the exact configuration of our RPC and MRPC prototypes. A picture of an MRPC module mounted inside such a gas chamber before it is sealed is shown in Fig. 4. In all tests, pure tetrafluoroethane gas (C_2_F_4_H_2_) was circulated through the detectors at a rate of a few liters per hour.

**Fig. 5.**
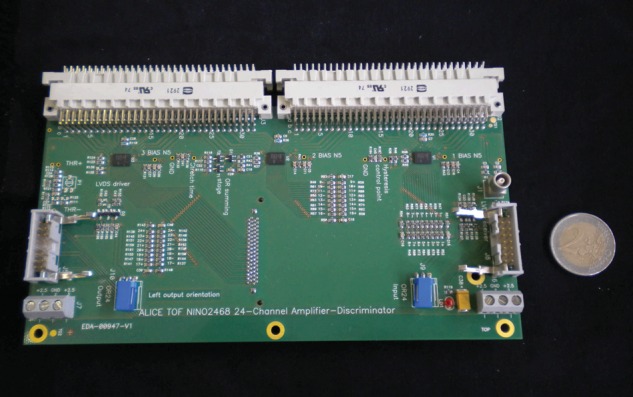
The 24-channel NINO card designed for the ALICE experiment and used in our tests.

Data acquisition was achieved with a front-end readout electronics board produced for the ALICE collaboration and based on the NINO multi-channel amplifier-discriminator ASIC [15]. The NINO produces Low Voltage Differential Signals (LVDS) with < 25-ps time jitter. Figure 5 shows a 24-channel ALICE TOF NINO front-end card (holding three NINOs) that has been used for our measurements.

**Fig. 6.**
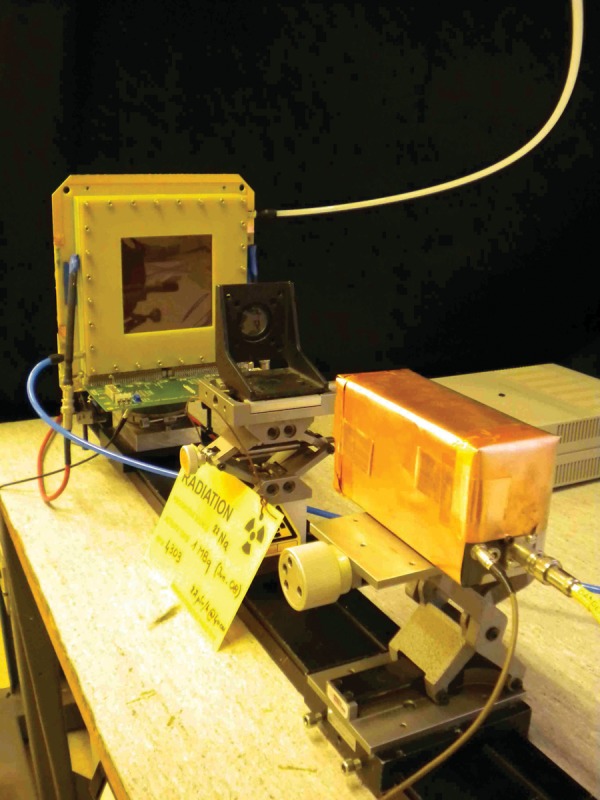
The experimental setup for RPC efficiency tests with 511-keV photons.

**Fig. 7.**
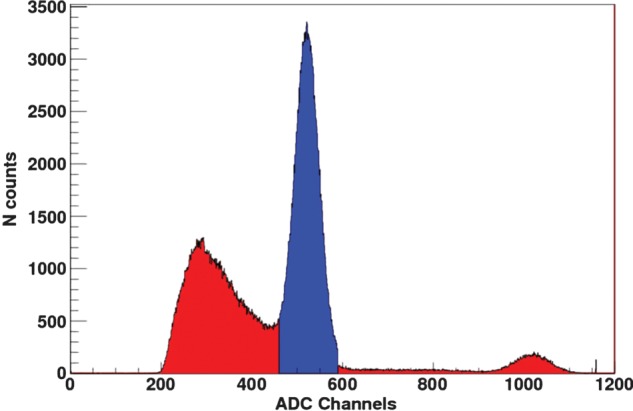
An example of the selection of photoelectric events in the BGO-PMT assembly used in the RPC efficiency measurements.

Our first task was to measure the efficiency of prototype single-gap and MRPC modules. To do so, we placed the prototypes opposite a 2.54 cm diameter BGO scintillator coupled to a photo-multiplier tube (PMT) with a ^22^Na point source positioned precisely between. Care was taken to ensure that, geometrically, all gamma rays detected by the crystal would have the corresponding co-linear gamma rays passing through the MRPC at the same time. The setup is shown in Fig. 6. Events within the crystal, digitized by an analog-to-digital converter (ADC), were selected to be within the photo-peak, as shown in Fig. 7. In this way, the efficiency was defined simply as the number of events counted in both the RPC and the BGO-PMT (coincidence within a narrow time window of about 50 ns) divided by the total number of events counted in the BGO, during a time interval of several minutes. The detection efficiency was measured as a function of the applied voltage, on prototypes having resistive layers of two different values of resistivity, 150 kΩ/square and 1 MΩ/square.

**Fig. 8.**
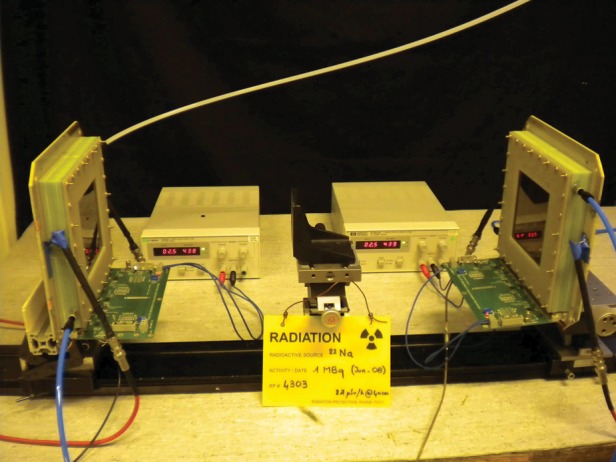
The experimental setup for RPC time-of-flight measurements with 511-keV photons.

For timing studies two identical MRPC modules were placed in coincidence about the ^22^Na source, as shown in Fig. 8. Two NINO front-end boards were used, one for each detector. The NINO's LVDS outputs were converted to NIM level with standard NIM electronics and fed to a CAMAC TDC (LeCroy 2228A) having 100-ps time resolution. The intrinsic timing characteristics of the electronics chain was measured by injecting charge into both detectors from a pulse generator simultaneously, and were found to be limited only by the resolution of the TDC itself.

## RESULTS

**Fig. 9.**
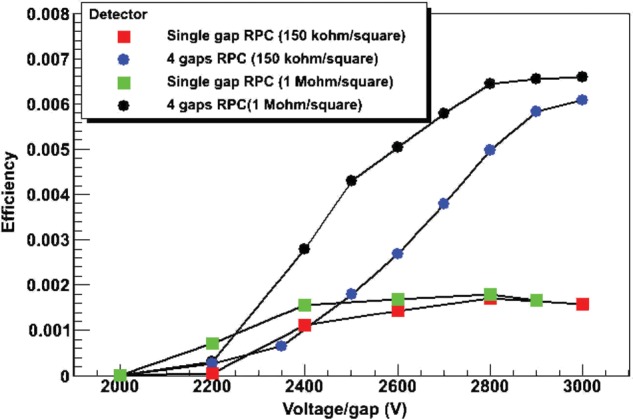
Single-gap and 4-gap RPC efficiency with 511-keV gamma rays. Two values of resistivity have been used for each configuration.

The efficiency of single-gap RPCs and 4-gap MRPCs, each with two different values of resistivity, has been measured as a function of the applied voltage per gap. Figure 9 shows the results for all four detector configurations. The efficiency of single-gap RPCs was found to reach a maximum at ∼ 0.18%, while for the 4-gap MRPCs the maximum was reached at ∼ 0.66%. In both cases, the plateau of efficiency was reached at lower voltages for the detectors having the higher resistivity.

**Fig. 10.**
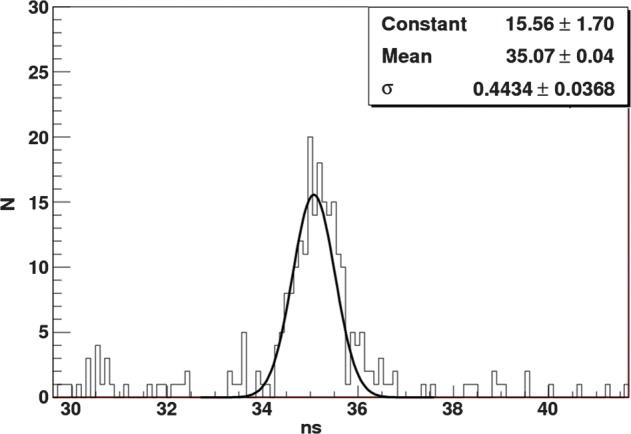
Time resolution of two single-gap RPC modules in coincidence with 511-keV gamma rays.

**Fig. 11.**
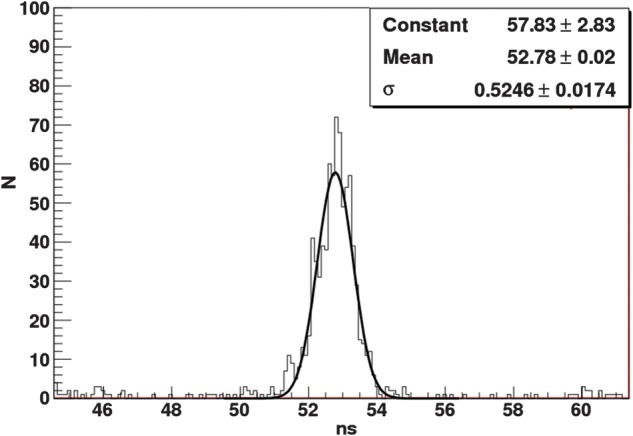
Time resolution of two 4-gap MRPC modules in coincidence with 511-keV gamma rays.

A preliminary result for the timing measurement for two single-gap RPC modules in coincidence is shown in Fig. 10. The result for two 4-gap MRPC modules is shown in Fig. 11. In both cases, resistive coatings of 1 MΩ/square were used. The standard deviation of the peak was 443 ps for the single-gap RPCs and 525 ps for the 4-gap MRPC modules. This translates into a single detector time resolution for 511 keV gamma rays of 310 ps and 370 ps for the single-gap and 4-gap modules, respectively.

## DISCUSSION

**Fig. 12.**
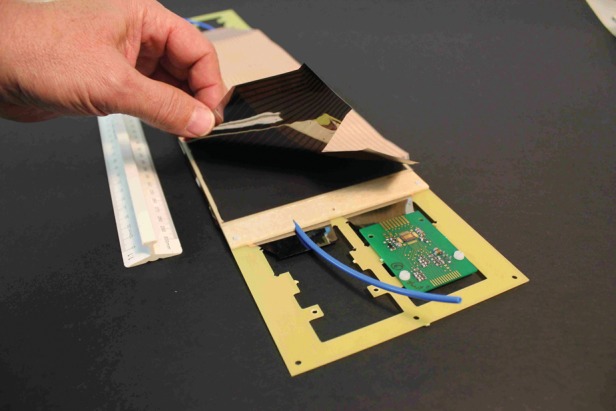
A 10 cm × 30 cm active area MRPC module destined for future testing. The new custom-built front-end electronics board based on the NINO is also shown.

We have developed a production technique for building compact MRPC modules that have been tested in the laboratory for gas tightness and HV stability. In addition to the 6.5 cm × 9 cm glued modules which have been developed, we have also recently produced our first glued 4-gap MRPC module on a scale suitable for a real PET scanner: 10 cm × 30 cm active area. One such MRPC module is shown in Fig. 12.

Using the same materials and geometry we have made efficiency tests and measured 0.18% and 0.66% for single and 4-gap RPC modules, respectively. These results are in line with simulations that have been carried out and reported by other groups [10]. Our own simulations, part of a larger study that is ongoing and not presented here, predict an efficiency of 0.78% for a 4-gap MPRC and 0.20% for single-gap.

On the other hand, the timing resolutions achieved for both the single-gap and MRPCs are significantly worse than the value demonstrated for MRPCs with charged particle detection. For the moment, our result of 310 ps and 370 ps single detector time resolution for the single-gap and 4-gap RPC modules, respectively, is not sufficient for an ibPET application. It must be mentioned, however, that our timing measurements have not been corrected for time-walk (slewing), which can introduce a significant uncertainty. This is because, depending on the amplitude of the analog signal, the exact moment that it crosses the threshold will vary; larger signals have shorter slewing times while smaller signals have longer slewing times.

For most NINO applications with RPCs, the time-walk correction is essential for achieving time resolutions of <100 ps FWHM. The correction is done by digitizing the time-over-threshold of the NINO output pulse (essentially its width), which is roughly proportional to the input charge or amplitude, and using this information to correct the time jitter introduced by slewing. So far, our attempts to observe a correlation between the TOF and the NINO output pulse width have been inconclusive, possibly due either to limitations in the electronics chain or to an insufficient signal-to-noise ratio, caused by the fact that only a single-gap is fired for each 511-keV detection.

At the time of writing, a custom-built electronics solution, also based on the NINO front-end amplifier/discriminator chip, but designed specifically for our glued and compact MRPC modules, is being debugged and is nearly ready for integration. In addition, we have developed a method of measuring more accurately the time resolution and the width of NINO outputs, which constitutes a great improvement to the electronics chain that has been used up until now. We are confident that with this new electronics solution we will be able to perform a time-over-threshold correction and achieve a better timing result.

Other aspects of the use of MRPCs, however, have been confirmed experimentally, namely the intrinsic efficiency of single-gap and multi-gap modules for 511-keV detection. In addition, a novel mechanical design has been implemented which has little excess mechanical material and which allows the construction of chambers that are easy to assemble in large quantity. This is an important point since any RPC-PET detector must consist of many hundreds of multi-gap modules so as to achieve a sensitivity comparable to crystal-based technology.

## FUNDING

This research has been supported by the Marie Curie Early Initial Training Network Fellowship of the European Community's Seventh Framework Programme under Contract No. PITN-GA-2008-215840-PARTNER.
